# Robust humoral and cellular immune responses in long-term convalescent COVID-19 individuals following one-dose SARS-CoV-2 inactivated vaccination

**DOI:** 10.3389/fimmu.2022.966098

**Published:** 2022-08-01

**Authors:** Boyun Liang, Tiandan Xiang, Hua Wang, Ziwei Li, Xufeng Quan, Xuemei Feng, Sumeng Li, Sihong Lu, Lei Fan, Ling Xu, Tong Wang, Xiaoyan Wang, Bin Zhu, Junzhong Wang, Dongliang Yang, Jia Liu, Xin Zheng

**Affiliations:** ^1^ Department of Infectious Diseases, Union Hospital, Tongji Medical College, Huazhong University of Science and Technology, Wuhan, China; ^2^ Joint International Laboratory of Infection and Immunity, Huazhong University of Science and Technology, Wuhan, China; ^3^ Department of Infectious Diseases, Shandong Provincial Hospital Affiliated to Shandong First Medical University, Jinan, China; ^4^ Department of Laboratory Medicine, Maternal and Child Health Hospital of Hubei Province, Tongji Medical College, Huazhong University of Science and Technology, Wuhan, China

**Keywords:** COVID-19, long-term convalescent, SARS-CoV-2 inactivated vaccine, immune responses, VOCs

## Abstract

COVID-19, caused by SARS-CoV-2, has resulted in hundreds of millions of infections and millions of deaths worldwide. Preliminary results exhibited excellent efficacy of SARS-CoV-2 vaccine in preventing hospitalization and severe disease. However, data on inactivated vaccine-induced immune responses of naturally infected patients are limited. Here, we characterized SARS-CoV-2 RBD-specific IgG (anti-S-RBD IgG) and neutralizing antibodies (NAbs) against SARS-CoV-2 wild type and variants of concerns (VOCs), as well as RBD-specific IgG-secreting B cells and antigen-specific T cells respectively in 51 SARS-CoV-2 recovered subjects and 63 healthy individuals. In SARS-CoV-2 recovered patients, a single dose vaccine is sufficient to reactivate robust anti-S-RBD IgG and NAbs. The neutralizing capacity against VOCs increased significantly post-vaccination no matter healthy individuals or SARS-CoV-2 recovered patients. In addition, RBD-specific IgG-secreting B cells in SARS-CoV-2 recovered patients were significantly higher than that in healthy vaccine recipients. After the vaccine booster, the frequencies of specific IFN-γ^+^ CD4^+^ T cell, IL-2^+^ CD4^+^ T cell, and TNF-α^+^ CD4^+^ T cell responses were significantly increased in SARS-CoV-2 recovered patients. Our data highlighted the safety and utility of SARS-CoV-2 inactivated vaccine and demonstrated that robust humoral and cellular immune response can be reactivated by one-dose inactivated vaccine in SARS-CoV-2 recovered patients.

## Introduction

The coronavirus disease 2019 (COVID-19), caused by severe acute respiratory syndrome coronavirus 2 (SARS-CoV-2) infection, has resulted in hundreds of millions of infections and great mortality worldwide. The increased transmission and pathogenicity of the emerging various SARS-CoV-2 variant of concerns (VOCs, Alpha, Beta, Gamma, Delta, and Omicron) further aggravate the persistence of the pandemic (1–3). Different SARS-CoV-2 vaccines are widely administered in healthy individuals worldwide to cope with the current epidemic situation (4–6). Preliminary results also exhibit excellent efficacy of the vaccine in preventing hospitalization and severe disease in healthy individuals (7, 8).

Natural SARS-CoV-2 infection induced durable antibody response and cellular immune memory at least 8-12 months in previous reports (9, 10), but the neutralizing antibody titers dropped to a relatively low level, especially neutralization to the VOCs (10–13). Hence, it is important to further explore the humoral and cellular immune response of boost vaccine on convalescent patients and investigate their capacity to neutralize various SARS-CoV-2 VOCs.

Several previous studies showed that the SARS-CoV-2 vaccines can prime robust humoral and cellular immune response in SARS-CoV-2 recovered patients and a single dose is sufficient to reactivate immune memory (14–17). However, most studies focused on mRNA vaccines. In addition, the patients enrolled in these studies had a relatively short interval (about 2-10 months) between symptom-onset and vaccination, and the immunological memory of these early convalescent COVID-19 patients was still maintained at a relatively high level (18, 19). To date, data on the safety and protective immunity of SARS-CoV-2 inactivated vaccination for long-term (more than one year) convalescent patients are still limited.

Here, we aimed to characterize the IgG antibody against the receptor-binding domain (RBD) of spike protein (anti-S-RBD IgG), as well as the neutralizing antibodies (NAbs) against the original SARS-CoV-2 (wild type, WT) and VOCs in long-term recovered patients (approximately 16 months after symptom onset) following SARS-CoV-2 inactivated vaccination. Furthermore, SARS-CoV-2 RBD specific B cells response and antigen-specific T cells response to SARS-CoV-2 overlapping peptides were investigated. The graphic abstract including two cohorts were provided in **Figure S1**. Evaluation of the safety and protective immunity of long-term recovered patients boosted with SARS-CoV-2 inactivated vaccines in the real world would lay a solid foundation for scientific epidemic prevention and provide a theoretical basis for vaccine optimization.

## Methods

### Study design and participants

51 SARS-CoV-2 recovered individuals (SR) who were pre-vaccinated or had completed the first or second dose of inactivated vaccines (CoronaVac, BBIBP-CorV, or WIBP-CorV) were consented and enrolled in our study. 63 healthy subjects(HC) with completed (two doses) vaccination were enrolled as controls. All the healthy donors (without known SARS-CoV-2 infection history) were recruited based on self-reported symptom-free with negative IgG antibody and negative PCR test of nasopharyngeal swab before vaccination. The disease severity of COVID-19 recovered patients was defined according to the Guidelines of the Diagnosis and Treatment for SARS-CoV-2 issued by the Chinese National Health Committee (Version 7). All participants were without severe health conditions or immune-related diseases that may affect the vaccine response. Clinical data in the acute phase were obtained from electronic medical records, including demographic, clinical manifestation, and comorbidities. The overall incidence of adverse reactions was collected by two trained physicians *via* a standard questionnaire. The study protocol was approved by the Ethics Committee of Wuhan Union Hospital, Tongji Medical College, Huazhong University of Science and Technology.

### Sample collection and isolation

5-10mL of venous blood from participants was collected to isolate plasma and peripheral blood mononuclear cells (PBMCs). After centrifugation at 3000g for 15 min, followed by 30 min inactivation at 56°C, plasma samples were stored at -80 °C for further experiments. PBMCs were isolated using Ficoll density gradient centrifugation (DAKEWE Biotech, Beijing, China). The fresh PBMCs were stimulated rapidly and then tested by flow cytometry analysis, and the remaining PMBCs were cryopreserved in cell freezing medium (NCM biotech, Suzhou, China).

### SARS-CoV-2 specific antibodies analysis

The anti-S-RBD IgG kit in an indirect chemiluminescence immunoassay to recognize the SARS-CoV-2 receptor binding domain (RBD) of the S protein. The SARS-CoV-2 Neutralizing Antibody Assay Kit (WT, Alpha, Beta, Delta, and Omicron) is an *in vitro* quantitation of ACE-2:RBD interaction by the fully-auto chemiluminescence immunoassay analyzer MAGLUMI™ X8 (Snibe, Shenzhen, China). The antibody titer in the blood is tested for the level of blockade of the interaction. The kit reported that anti-S-RBD-IgG tests have 100% sensitivity(≥15 days post symptom onset) and 99.6% specificity for the diagnosis of COVID-19. The cut-off value was 1 AU/mL for anti-S-RBD-IgG and 5 AU/mL for NAbs. The specific antibody detection methods were performed as previously described (20).

### Enumeration of B cells secreting IgG antibodies specific for the SARS-CoV-2 RBD

The Enumeration of B cells secreting SARS-CoV-2 RBD-specific IgG was measured by ELISpot Path: Human IgG (SARS-CoV-2, RBD) HRP kit (Mabtech AB, Sweden) as manufacturer’s procedure. Briefly, cryopreserved PBMCs were resuscitated and rested in a 37°C and 5% CO_2_ incubator. Then, cells were pre-stimulated *in vitro* with complete medium containing R848 (1 µg/ml) and rIL-2 (10 ng/ml) for three days under sterile conditions to secrete a detectable amount of antibody (21). Following pre-stimulation, the cells were incubated in an ELISpot plate for an additional 24 hours. Washed the plate extensively, added 100ul/well diluted RBD-WASP solution, and incubated for 2h at room temperature. Anti-WASP-HRP solution and TMB substrate solution were subsequently added; developed until spots emerged, stopped, and left the plate to dry. The spots were inspected and counted using an automated ELISpot reader.

### Analysis of the SARS-CoV-2-specific T cell response

#### Virus-specific T cells stimulation

Fresh PBMCs suspension was seed on 96-well flat-bottom plate. Prepare the mixture of complete medium (RPMI-1640 containing 10% fetal calf serum, 100U/ml penicillin, 100ug/ml streptomycin, and 100um HEPES) with or without overlapping peptide pools covering entire sequences of SARS-CoV-2 spike glycoprotein (S, GenScript, Cat No.RP30027), membrane (M, GenScript, Cat No.RP30022), or nucleocapsid (N, GenScript, Cat No.RP30013) proteins respectively. Then costimulatory reagent anti-CD28/CD49d (BD Biosciences) and recombinant interleukin-2 (rIL-2; Hoffmann-La Roche) were added to the cultures. The PBMCs were stimulated for 9 days *in vitro* at a 37°C and 5% CO_2_ incubator, with fresh medium containing IL-2 added twice a week. On day 10, the cells were harvested and tested for intracellular expression after re-stimulation with corresponding peptide pools for 5 hours. Polyclonally activators (PMA, phorbol 12-myristate 13-acetate and Iono, ionomycin), were added as positive controls and those without peptide stimulation as negative controls. Brefeldin A (BFA, eBioscience, Invitrogen™ USA) was added to block cytokine release outside the cell. After re-stimulation, the cells were analyzed by intracellular cytokine staining (ICS) with subsequent flow cytometry.

#### Surface and intracellular cytokine staining

For cell surface staining, cells were incubated with relevant fluorochrome-labeled antibodies for 30 min at 4°C in the dark. The anti-human monoclonal antibodies used in surface staining included APC-eFluor^®^ 780-anti-CD3, PerCP-Cyanine5.5-anti-CD4, and PE-Cyanine7-anti-CD8 (all from eBioscience, Invitrogen™, USA). Cell debris and dead cells were excluded from the analysis based on scatter signals and Fixable Viability Dye eFluor™ 506 (eBioscience, Invitrogen™, USA). For ICS, cells were stained with FITC-anti-IFN-γ, PE-anti-IL-2, and APC-anti-TNFα (all from eBioscience, Invitrogen™, USA) after fixed and permeabilized with the Intracellular Fixation & Permeabilization Buffer Set (Invitrogen, USA). Approximately 100,000 events were acquired for each sample using a BD FACS Canto II flow cytometer. Data were processed using Flow Jo software V10.0.7 (Tree Star, Ashland, OR, USA). For data analysis, frequencies of cytokines-producing CD4+ and CD8+ T cells were analyzed by subtracting the unstimulated control (UN). T cell response was defined as positive expression exceeding 2-fold UN. Additionally, the number of peptide pools the T cells responding to was defined as the breadth of SARS-CoV-2-specific T cell responses. And, the number of producing-cytokines was defined as the functionality of SARS-CoV-2-specific T cell responses. Samples with non-response positive controls were excluded from further analysis.

### Statistical analysis

Statistical significance was determined by SPSS software (version 25.0, IBM, Chicago, IL, USA) and graphical presentations were made with GraphPad Prism (version 9.0, California, USA) or Origin (version 2022, Massachusetts, USA). Continuous variables were presented as median and interquartile range (IQR), while categorical variables were expressed as number (n) and percentage (%). The Chi-square or Fisher’s exact test was used for categorical data statistical analysis. Paired or unpaired t-test, Mann-Whitney U test, and Kruskal-Wallis test followed by Dunn’s multiple comparisons test were used to determine significant differences for continuous data where appropriate. Correlations between two continuous variables were performed by Spearman’s rank correlation test. A two-sided P value <0.05 was considered statistically significant. ns, no significance; *, *P*<0.05; **, *P*<0.01; ***, *P <*0.001; ****, *P <*0.0001.

## Results

### Characteristics of the study cohort

A total of 149 samples (SR: N=80 and HC: N=69) from 114 participants (SR: N=51 and HC: N=63) were collected and divided into four groups based on previous COVID-19 exposure and vaccination: (1) HC-baseline: healthy individuals without vaccination, (N=14); (2) HC-vaccination: healthy individuals with vaccination, (N=55); (3) SR-baseline: SARS-CoV-2 recovered individuals without vaccination, (N=40); (4) SR-vaccination: SARS-CoV-2 recovered individuals with vaccination, (N=40) (**Figure S1**). 55 participants from the HC-vaccination group (55 samples) all received two doses of inactivated vaccines and 6 participants had sequential samples pre- and post-vaccination. In addition, the 40 samples of the SR-vaccination group were collected from 35 SARS-CoV-2 recovered patients, containing 30 samples following one-dose immunization and 10 samples following two-dose immunization. In 24 SR participants, blood samples were collected at 2 to 4 different time points pre- and post-vaccination. The demographic and vaccine-related information of the recruited participants is provided in **Table S1**. The median time from symptom onset to follow-up in the SR-vaccination group was 549 days (IQR:518-592 days); the IQR of interval time from 1st vaccination to follow-up ranged from 21 to 65.5 days with a median time of 44 days.

### Safety evaluation of inactivated vaccines in SARS-CoV-2 recovered participants

**Figure 1:**
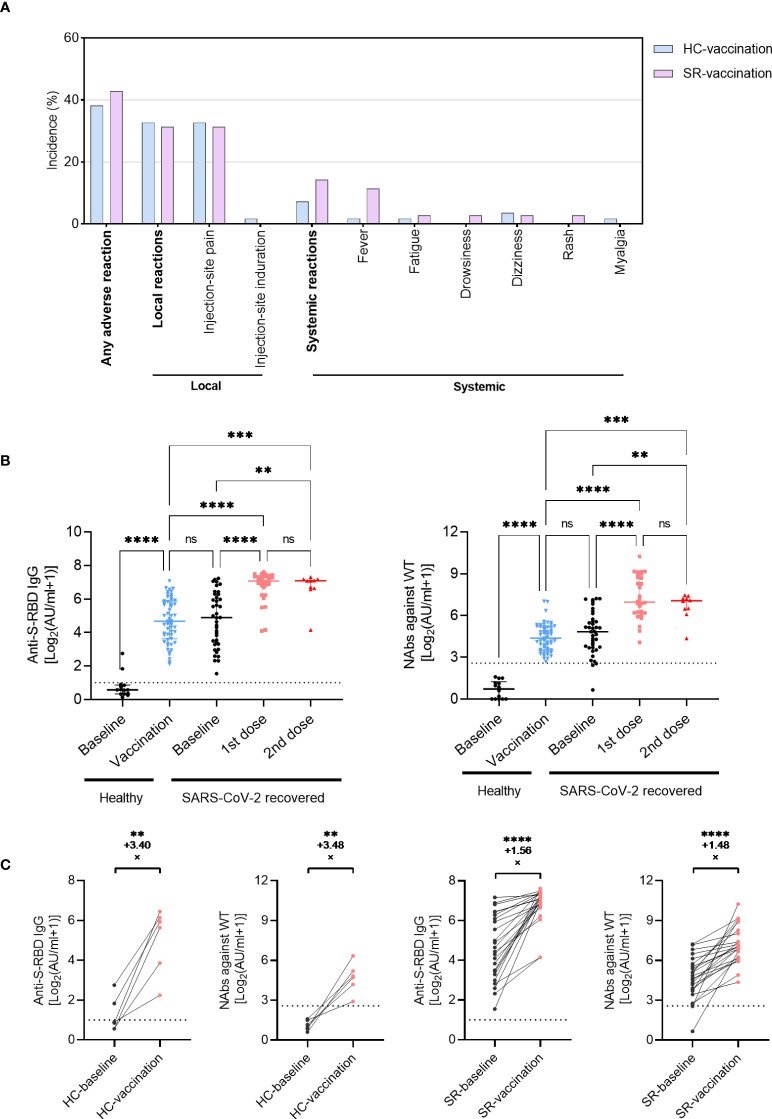
Antibody responses following inactivated vaccination in SARS-CoV-2 recovered patients (SR) and healthy individuals (HC). **(A)** The incidence of adverse reactions after vaccination in SR (N=35) and HC (N=55) patients. **(B)** The titer of anti-S-RBD-IgG and NAbs (WT) before or after vaccination in SR (baseline, N=40; vaccination, N=40 with 1st dose, N=30; 2nd dose, N=10) and HC (baseline, N=14; vaccination, N=55) group. Data are shown with median and IQR. **(C)** The fold-change of anti-S-RBD-IgG and NAbs increased pre- (black) and post- (red) vaccination in paired HC (N=6, paired data) and SR (N=24, paired data) individuals. The horizontal dotted line or shadow represented the cut-off value. The Kruskal-Wallis test followed by Dunn’s multiple comparisons test **(B)** and paired t-test **(C)** were used for statistical analysis. ns, no significance, **P<0.01, ***P <0.001, ****P < 0.0001.

The adverse reactions were investigated from vaccination recipients *via* a standard questionnaire to evaluate the safety of inactivated vaccines. The overall incidence of adverse reactions was 42.9% in 35 SARS-CoV-2 recovered individuals (**Figure 1A**; **Table S2**). Injection-site pain and swelling were the most common adverse reactions with an incidence of 31.4%, which was comparable to that in the HC-vaccination group (32.7%). For systematic adverse reactions, 11.4% (4/35) SR participants had a mild fever following vaccination and only 1.8% (1/55) in the HC-vaccination group (*P*=0.073, **Table S2**). There was no serious side effect in our investigation and all of the adverse reactions were mild and self-resolved consistent with previous reports (17).

### Antibody responses to SARS-CoV-2 inactivated vaccines

At baseline, all healthy individuals had undetectable levels of NAbs against WT, while almost all SARS-CoV-2 recovered patients had positive antibodies over one-year post symptom onset, only 3 individuals with NAbs against WT below the cut-off value (**Figure 1B**). Following two doses of vaccines, the anti-S-RBD IgG and NAbs against WT boosted significantly in healthy individuals up to a comparable level of antibody titers as SARS-CoV-2 exposed participants (**Figure 1B**; anti-S-RBD IgG, *P*<0.001; NAbs against WT, *P*<0.001). Similar changes were observed in 6 healthy individuals who had pre- and post-vaccination longitudinal paired data with an increased fold-change of 3.40 in anti-S-RBD IgG and 3.48 in NAbs against WT (**Figure 1C**). In SARS-CoV-2 recovered patients, both anti-S-RBD IgG and NAbs against WT increased rapidly following the first dose vaccination, whereas no significant increase of antibody titers was observed after the second vaccination (**Figure 1B**). All the 24 recovered participants with pre- and post-vaccination longitudinal paired data expressed positive responses of anti-S-RBD IgG and NAbs against WT, even in one female recipient with undetectable NAbs before vaccination (from 0.58AU/ml to 101.1AU/ml). The anti-S-RBD IgG increased with a fold-change of 1.56 in the SR group, and the NAbs increased with a fold-change of 1.48 (**Figure 1C**). The increased magnitude of antibody titer in recovered patients was smaller than that in healthy individuals.

### Neutralizing antibodies against SARS-CoV-2 VOCs after inactivated vaccination

**Figure 2:**
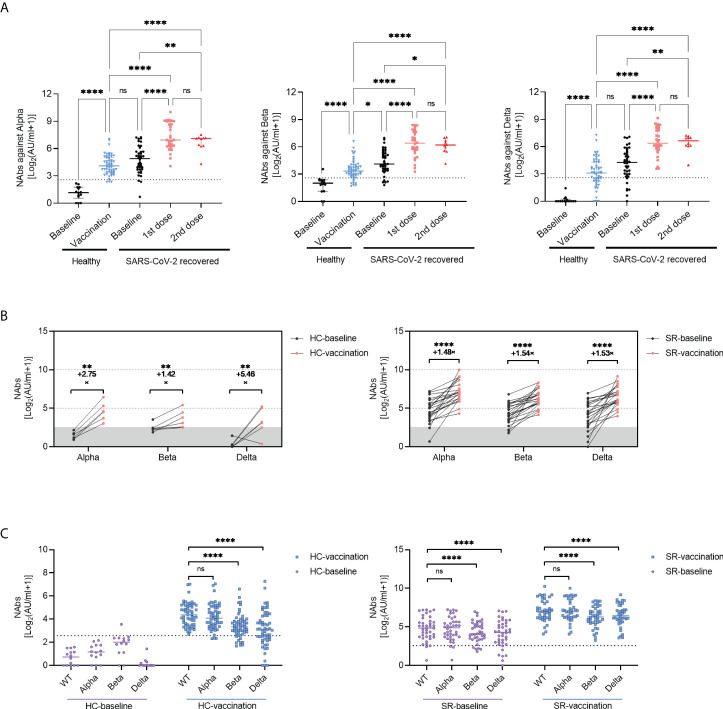
Neutralizing Antibody against variant of concerns (VOCs) following inactivated vaccination. **(A)** The titers of NAbs (VOCs) before or after vaccination in SR (baseline, N=40; vaccination, N=40 with 1st dose, N=30; 2nd dose, N=10) and HC (Baseline, N=14; vaccination, N=55) group. Data are shown with median and IQR. **(B)** he changes of NAbs against VOCs increased pre- (black) and post- (red) vaccination in paired HC (N=6, paired data) and SR (N=24, paired data) individuals. **(C)** he comparison of NAbs titers between SARS-CoV-2 wild type(WT)and VOCs in HC (left) and SR (right) group. The horizontal dotted line or shadow represented the cut-off value. Kruskal-Wallis test followed by Dunn’s multiple comparisons test (A, C) and paired t-test **(B)** were used for statistical analysis. ns: no significance, *P < 0.05, **P<0.01, ****P < 0.0001. HC, healthy control; SR, SARS-CoV-2 recovered patients.

Focusing on the emergence of VOCs, such as B.1.1.7 (Alpha), B.1.351 (Beta), and B.1.617.2 (Delta) exhibited immune evasion capacity, we further assessed the neutralizing antibodies against the VOCs using sera from healthy and SARS-CoV-2 recovered vaccine recipients. The results showed that the neutralizing capacity against VOCs increased significantly after vaccination no matter in healthy individuals or SARS-CoV-2 recovered individuals (**Figure 2A**). Besides, the increasing trend of vaccine-induced neutralizing antibodies against VOCs was similar to anti-S-RBD IgG and NAbs against wild type (WT), we didn’t observe additional neutralizing antibody responses against VOCs in SARS-CoV-2 recovered individuals after the second dose of vaccine (**Figure 2A**). In longitudinal paired data, the NAbs titers against VOCs of 6 HC and 24 SR individuals had varying degrees of elevation (**Figure 2B**). Encouragingly, SARS-CoV-2 exposed patients with undetectable NAbs against VOCs over one-year post symptom onset performed excellent neutralization activity following enhanced immunization with COVID-19 inactivated vaccine (**Figure 2B**, right). Moreover, **Figure 2C** illustrated that compared with the NAbs titers against WT, the titers against B.1.351 (Beta), and B.1.617.2 (Delta) VOCs decreased both in SR and HC groups, regardless of vaccination (all *P*<0.0001). There was no significant difference of NAbs titer between wild type and B.1.1.7 (Alpha) VOCs (**Figure 2C**). In addition, no significant difference of anti-S-RBD IgG and NAbs titers was observed between different age, sex, and disease severity in SARS-CoV-2 recovered patients (**Figure S2**).

**Figure 3:**
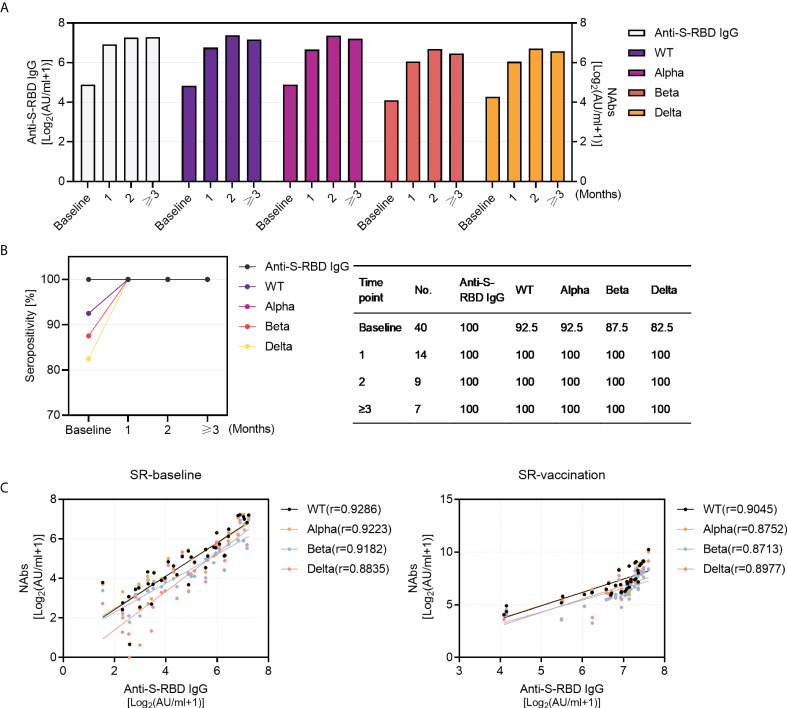
Kinetics of antibody responses in SARS-CoV-2 recovered patients with inactivated vaccination and the correlation between anti-S-RBD IgG and NAbs. **(A)** The dynamics of antibody titers (left Y-axis: anti-S-RBD IgG; right Y-axis: NAbs titers against WT and VOCs) within 3 months in SARS-CoV-2 recovered patients (baseline, N=40; post-vaccination 1 month, N=14, 2 months, N=10, and ≥3 months, N=16). **(B)** Sero-positivity within 3 months in SARS-CoV-2 recovered patients (baseline, N=40; post-vaccination 1 month, N=14, 2 months, N=10,and ≥3 months, N=16). **(C)** The correlation between anti-S-RBD IgG and NAbs pre- (N=40) and post- (N=40) vaccination in SARS-CoV-2 recovered group. Spearman’s rank correlation test was used for statistical analysis.

Next, we investigated the longitudinal profiling of humoral immune response over time. The results demonstrated robust antibody responses to SARS-CoV-2 wild type and VOCs last for at least 3 months and remained at a relatively stable level after COVID-19 inactivated vaccination (**Figure 3A**), with the longest data of 115 days post-vaccination in this study. Similarly, the seropositivity of NAbs against WT and VOCs remained stable (100%) in 3 months after vaccination (**Figure 3B**). Considering that anti-spike IgG played a crucial role in neutralization against SARS-CoV-2 infection, we further analyzed the relationship between anti-S-RBD IgG and NAbs. The titers of anti-S-RBD IgG correlated strongly with NAbs titers against SARS-CoV-2 WT and VOCs in SR individuals no matter pre- or post-vaccination (**Figure 3C**).

**Figure 4:**
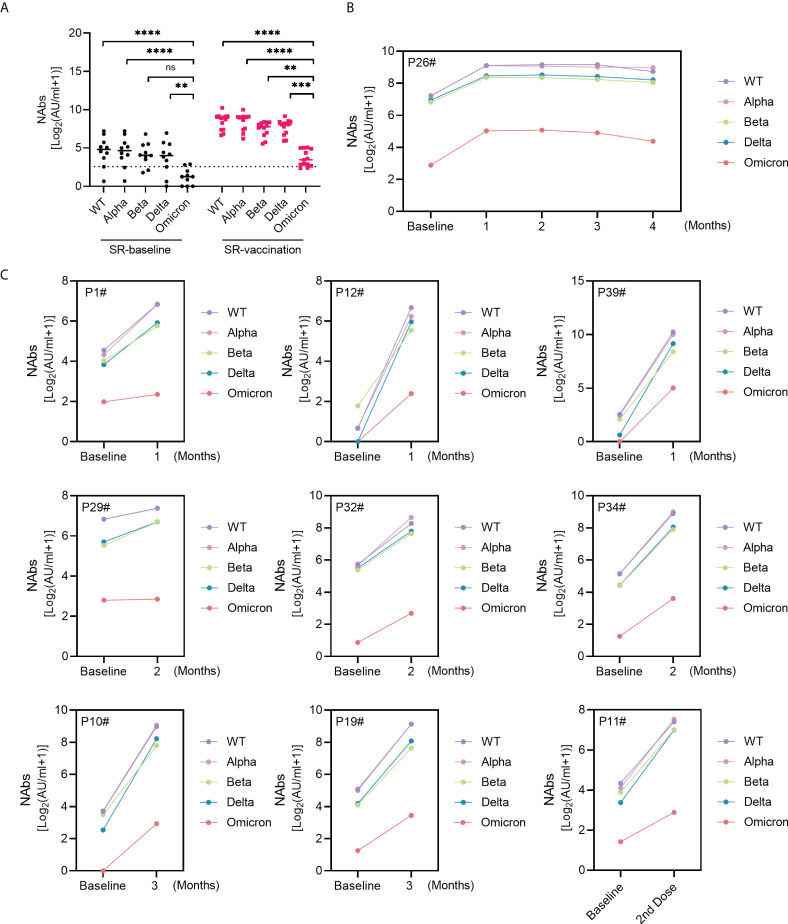
NAbs against Omicron in SARS-CoV-2 recovered patients following inactivated vaccination. **(A)** The comparison of NAbs between Omicron variant and WT or VOC. **(B, C)** Sequential response of NAbs against Omicron and other virus types in 10 SARS-CoV-2 recovered patients. Kruskal-Wallis test was used for statistical analysis. ns: no significance, **P<0.01, ***P <0.001, ****P < 0.0001.

The recent emerging VOC B.1.1.529 (Omicron) with more transmissible and immune escape has quickly raised global attention due to its multiple mutations. NAbs against Omicron were plotted in 10 SARS-CoV-2 recovered individuals in **Figure 4**. Obviously, Omicron variant showed the most significant resistance to neutralizing antibodies than other SARS-CoV-2 variants (**Figure 4A**). Notably, the plasma from SARS-CoV-2 exposed patients exhibited significant boost neutralizing ability to Omicron variant post enhanced vaccination (**Figures 4B, C**). NAbs against Omicron responded rapidly within one-month following one-dose vaccination and lasted for at least four months in one recovered patient (**Figure 4B**). A similar trend was observed in another 9 SR individuals; the titer of NAbs against Omicron within the first month, second month or third month was higher than that post-vaccination (**Figure 4C**).

### SARS-CoV-2 RBD-specific antibody-secreting cells (ASCs) responses to inactivated vaccines

**Figure 5:**
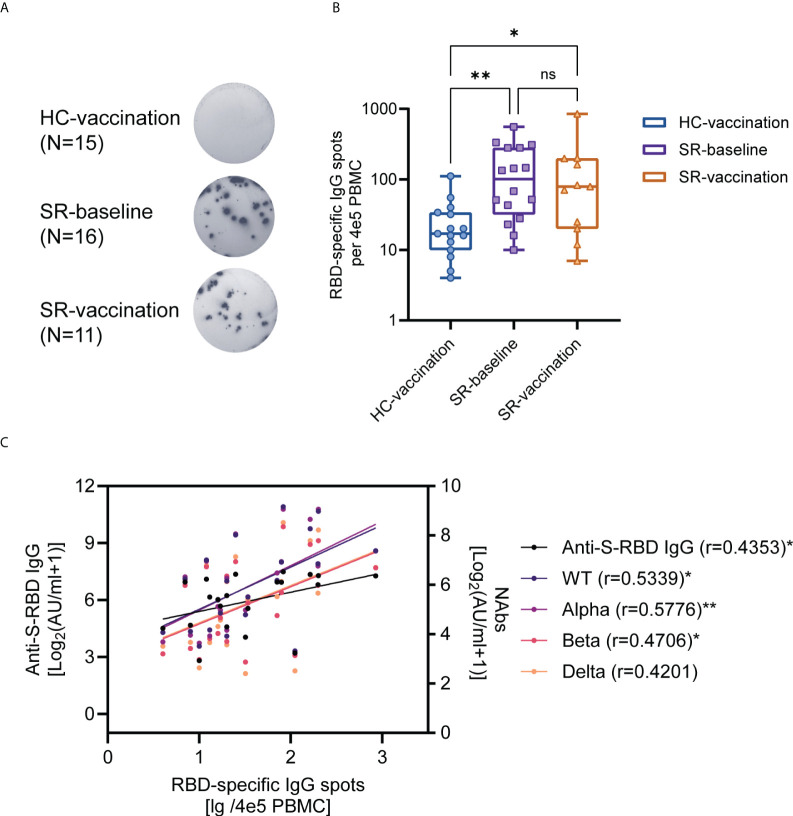
SARS-CoV-2 RBD-specific B cell responses after booster vaccination. **(A)** ELISpot assay for representative RBD-specific B cell spots in HC (Vaccination, N=15) and SR (Baseline, N=16; vaccination, N=11) subjects. **(B)** The comparison of the number of RBD-specific ASCs between SR patients and HC individuals. **(C)** The correlation between RBD-specific ASCs and antibody titers post-vaccination (N=21, HC-vaccination 10 samples, SR-vaccination 11 samples). Kruskal-Wallis test followed by Dunn’s multiple comparisons test **(B)** and Spearman’s rank correlation test **(C)** were used for statistical analysis. ns: no significance, *P < 0.05, **P<0.01, HC: healthy control; SR: SARS-CoV-2 recovered patients. ASCs: antibody-secreting cells. The median (IQR) number of RBD-specific ASCs for HC-vaccination group is 17(10-34), for SR-baseline group is 101(32-280), and for SR-vaccination group is 79(20-198).

To identify the function of SARS-CoV-2-specific B cells after vaccination, RBD-specific IgG ASCs were detected by ELISpot assay among HC-vaccination (N=15, completed vaccination), SR-baseline (N=16, without vaccination), and SR-vaccination (N=11, with one-dose vaccination) group (**Figure 5A**). RBD-specific IgG ASCs existed at pre-vaccination baseline in SARS-CoV-2 recovered individuals over one-year post symptom onset (**Figures 5A, B**). Nonetheless, the number of RBD-specific IgG ASCs whether at baseline (*P*<0.01) or post-vaccination (*P*<0.05) from the SARS-CoV-2 recovered group were significantly higher than that in healthy vaccine recipients, these pre-existing RBD-specific IgG ASCs were not further boosted after vaccination in our study (**Figure 5B**). Moreover, the number of spots of RBD-specific ASCs were also correlated with anti-S-RBD IgG (r=0.4353) and neutralizing antibody (against WT, r=0.5339; Alpha, r=0.5749; Beta, r=0.4592; Gamma, r=0.4670) titers (**Figure 5C**).

### Specific T cell responses to SARS-CoV-2 inactivated vaccines

To evaluate the effector capacity of SARS-CoV-2 specific T cells after vaccination, we stimulated the PBMCs *in vitro* with overlapping peptides spanning the SARS-CoV-2 spike (S) membrane (M), and nucleocapsid (N) proteins, and next characterized the multi-specific CD4^+^ and CD8^+^ T cells responses for intracellular cytokines (IFN-γ, IL-2, and TNF-α) production by flow cytometry. 12 SARS-CoV-2 recovered participants who received one-dose vaccine and 20 SARS-CoV-2 recovered participants without vaccination (baseline) were performed T cells detection. **Figure S3** showed the gating strategy of flow cytometry for intracellular cytokines production of CD4^+^ and CD8^+^ T cells.

**Figure 6:**
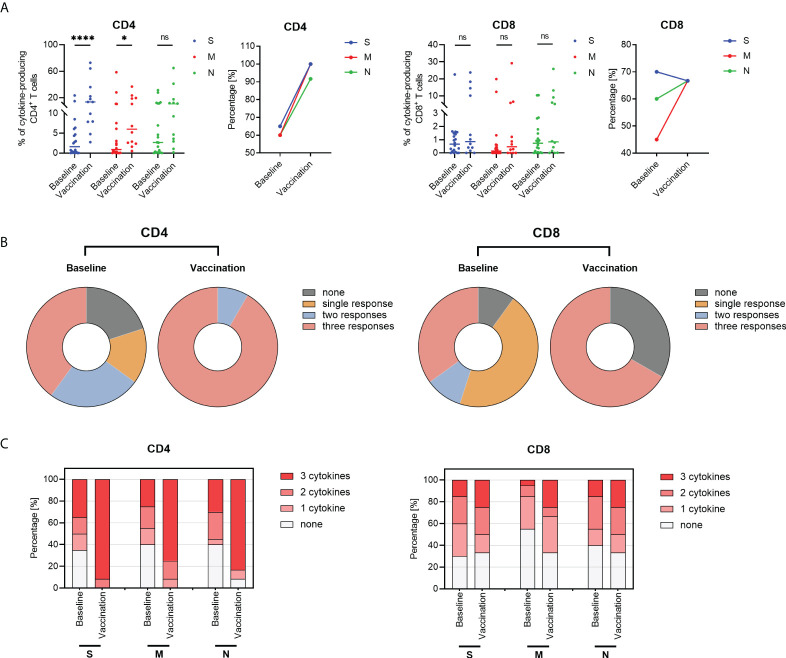
SARS-CoV-2-specific cytokine-producing T cells responses in SARS-CoV-2 recovered (SR) patients. **(A)** The frequencies and positive response rate of overall SARS-CoV-2-specific CD4^+^ T cells in responses to overlapping peptide pools including spike (S, blue), membrane (M, red), and nucleocapsid (N, green) peptide in SARS-CoV-2 recovered individuals at baseline (n=20) and post-vaccination (n=12). Each dot indicates one subject and the short line represents the median value of each group. **(B)** The breadth of SARS-CoV-2-specific CD4^+^ and CD8^+^ T cell responses at baseline and post-vaccination. **(C)** The multi-functionality of CD4^+^ and CD8^+^ T cell responses in SARS-CoV-2 recovered patients at baseline and post-vaccination. Frequencies of cytokines-producing CD4^+^ and CD8^+^ T cells were calculated as the background (UN, unstimulated control) subtracted. The magnitude of overall T cell response refers to the sum of the frequencies of any cytokine (IFN-γ, IL-2, and TNF-α) with peptide pool stimulation. A T cell response was defined as a positive expression exceeding 2-fold UN. The number of peptide pools the T cells responding to was defined as the breadth of SARS-CoV-2-specific T cell responses. The number of producing-cytokines was defined as the functionality of SARS-CoV-2-specific T cell responses. Samples with non-response positive controls were excluded from further analysis. Kruskal-Wallis test followed by Dunn’s multiple comparisons test was used for statistical analysis. ns: no significance, *P < 0.05, ****P < 0.0001.

In line with the findings of SARS-CoV-2-specific B cells, the circulating antigen-specific CD4^+^ and CD8^+^ T cells were still present in SARS-CoV- recovered subjects before vaccination (**Figure 6**). Firstly, we found that the magnitude of overall CD4^+^ T cell response which produced at least one cytokine (IFN-γ, IL-2, and TNF-α) with peptide pool stimulation was significantly higher after booster vaccination; and the S-, M-, and N-specific CD4^+^ T cell responses were detected in 100% (12/12), 100% (12/12), and 91.7% (11/12) of SR individuals post-vaccination (**Figure 6A**). Moreover, 66.7% (8/12) of SR individuals exhibited positive CD8^+^ T cell response post-vaccination. For the breadth of SARS-CoV-2-specific T cell responses, we observed that 20% (4/20) of the SR participants weren’t with any detectable specific CD4^+^ T cell responses at baseline, and 40% (8/20) showed T cell responses against three peptide pools. After boost vaccination, all the participants obtained specific CD4^+^ T cell responses against S, M, or N peptide pool, of which 91.7% (11/12) responded to three peptide pools (**Figure 6B**, left). At the baseline, CD8^+^ T cell responses against three peptide pools were detectable in 35% (7/20) of SR individuals and up to 66.7% (8/12) post-vaccination (**Figure 6B**, right).

Furthermore, similar results were illustrated when cytokine-producing T cell was analyzed as single cytokine expression. After vaccine immunization, the frequencies of specific IFN-γ^+^ CD4^+^ T cells, IL-2^+^ CD4^+^ T cells, and TNF-α^+^ CD4^+^ T cells in response to S peptides were significantly increased in SARS-CoV-2 recovered participants (**Figure S4**, all P<0.0001). In addition, IL-2^+^ CD4^+^ T cells in response to M and N peptides after vaccination were significantly higher than pre-vaccination in SARS-CoV-2 recovered participants. In contrast to CD4^+^ T cells, multi-specific CD8^+^ T cell responses appeared little augmented in SARS-CoV-2 recovered individuals post-vaccination (**Figure S4A**). Except for the frequencies of IFN-γ^+^ CD8^+^ T cells against spike antigen increased following immunization, there was no significant increase after stimulation with M or N peptides (**Figure S4**). We then focused on the multi-functionality of SARS-CoV-2-specific T cells producing multiple cytokines after vaccination. We observed increased frequencies of antigen-specific T cells producing 2 or 3 cytokines in combination with booster vaccination (**Table S3**). At baseline, more than half the individuals produced only one cytokine or none at all (**Figure 6C**; **Table S3**). After vaccination, the proportion of the multi-specific CD4^+^ T cells secreting three cytokines increased significantly (S, 91.7%; M, 75.0%; N, 83.3%; all *P*<0.05; **Figure 6C** left, **Table S3**). Although there was no statistical significance, a similar tendency of CD8^+^ T cells producing three-cytokines was observed following booster vaccination (**Figure 6C** and **Table S3**).

## Discussion

Heated debate has occurred about whether SARS-CoV-2 vaccination is necessary to recovered COVID-19 individuals who acquired protective immunity from natural SARS-CoV-2 infection (22, 23). Here, we provided comprehensive profiling of adaptive immune responses from COVID-19 recovered patients following SARS-CoV-2 inactivated vaccine. Our findings suggested that SARS-CoV-2 inactivated vaccines had a favorable safety profile in COVID-19 recovered individuals, and one single dose of inactivated vaccine was sufficient to elicit a robust antibody response. A booster vaccination could improve their capacity to neutralize SARS-CoV-2 WT and various VOCs (including Alpha, Beta, Delta, and Omicron). RBD-specific IgG secreting B cells were detectable in SARS-CoV-2 recovered patients, and significantly higher than that of healthy vaccine recipients. Moreover, antigen-specific T cell response exhibited more augmented magnitude and multi-functionality after vaccination. This real-world study filled the gap of limited data in clinical trials regarding SARS-CoV-2 recovered patients, providing different perspectives on the safety and immunogenicity of recovered patients receiving inactivated vaccines.

Our serological data are in agreement with several recent published studies about mRNA vaccines (14–17), indicating the maximized titers of anti-S-RBD IgG and neutralizing antibody were induced by one-dose vaccine. This may be due to the fact that the naturally infected individuals have a pre-existing SARS-CoV-2 specific memory response, so the first dose of vaccine plays a role as boost vaccination (9). In line with these results, healthy individuals only achieve a robust antibody response until they complete the two-dose vaccination regimen (14, 15). In addition, our data showed that all recipients’ ability to neutralize the SARS-CoV-2 VOCs significantly enhanced after vaccination. These results were encouraging and further illustrated the necessity for booster vaccination in the context of the prevalence of mutant strains. Notably, recent real-world studies proved that the vaccination among individuals with previous SARS-CoV-2 infection decreased the risk of reinfection and hospitalization (24, 25).

Data in this study was consistent with previous reports that no additional increase in antibody levels was observed after the second vaccine dose among SARS-CoV-2 recovered patients (15, 16, 26, 27). One possibility is that the high titer of antibodies after the first vaccine dose in SARS-CoV-2 recovered patients were sufficient to bind and prevent the presentation of spike epitopes, and the hyper-immune activation limits further boost of the immune response (26–28). Despite the second dose doesn’t show an additional increase of antibody titers in SARS-CoV-2 recovered individuals, whether it plays a role in other immunological features remains unclear. After all, Thiago et al. have found that a second dose of booster vaccination conferred additional protection contributing to reduced morbidity and mortality (24). Moreover, a longer dosing interval between two vaccinations and heterologous SARS-CoV-2 booster vaccination might favor better immune responses (20, 29). Thus, strategies regarding the second dose of SARS-CoV-2 vaccine in convalescent patients need to be further investigated.

As our expectation, the number of RBD IgG-secreting B cells by the recovered patients is significantly higher than that of healthy vaccine recipients, regardless of whether the recovered patients were vaccinated or not. It is understandable as SARS-CoV-2 recovered patients had pre-existing immunity before vaccination (30). Unexpectedly, the recovered patients showed no statistical difference in their specific B cell levels after vaccination compared with baseline levels. This was inconsistent with studies about memory B cells induced by mRNA vaccines, which resulted in a significant increase in RBD-specific memory B cells in SARS-CoV-2 recovered patients (14, 31). One possible explanation for this discrepancy is our baseline data were obtained from SARS-CoV-2 recovered subjects approximately 6 months earlier before vaccination. During this period, the pre-existing RBD-specific circulating B cells in the SARS-CoV-2 recovered group may experience a further contraction. Moreover, specific B cells are undergoing development and differentiation in the early phase of immune responses (21); the B cells response may not increase to the peak value at our following time. Anyway, our results exhibited favorable SARS-CoV-2-specific B cell response in SARS-CoV-2 recovered patients.

SARS-CoV-2 specific T cells constitute an important part of adaptive immunity against virus infection and correlate with clinical protection (32). Most naturally infected patients have established broad and strong T cell memory to multiple target SARS-CoV-2 antigen in the early convalescent phase, and the memory response declined over time with a half-life of 3-5 months (9, 19, 33). One-dose mRNA vaccination has been proved to reactivate robust SARS-CoV-2 specific memory T cell responses in COVID-19 recovered subjects. Our results demonstrated that strong CD4^+^ T cell and weak CD8^+^ T cell responses were reactivated by inactivated vaccination in COVID-19 recovered individuals over one year from disease onset, which may be due to the role of helper T (Th) cells assisting with antibody production. Similarly, a previous study about inactivated vaccines did not report strong CD8^+^ T cell responses (20). However, we didn’t observe significant correlation between the antibody titers and T cell responses (data unshown), consistent with the results in healthy individuals who received inactivated vaccination (5). In this regard, humoral and cellular immune responses in COVID-19 recovered individuals following SARS-CoV-2 inactivated vaccination should be further addressed in the future.

Our study has several limitations. First, further studies with larger samples are needed to identify the long-term immune memory after vaccination and the protective ability against re-infection. Second, it’s hard to acquire enough samples to characterize other phenotypical features of the SARS-CoV-2 specific lymphocytes, like follicular helper T cell (Tfh) and their correlation with antibody responses. Third, despite we have showed NAbs against Omicron in several individuals, it is necessary to further investigate the immune escape of emerging Omicron and other VOCs in larger cohort, which is currently responsible for most cases and deaths around the world (3). Lastly, the SARS-CoV-2 live virus or pseudo-typed neutralization assays is necessary to use in our future research.

Taken together, SARS-CoV-2 recovered patients expressed excellent humoral and cellular immune responses after being boosted by a single dose of inactivated vaccine. Thevaccination enhanced the protective effects of neutralizing antibodies against the VOCs in both SARS-CoV-2 infected or naive individuals. Our study helps determine the optimal strategies for vaccination. Given the continuous emergence of novel VOCs, booster vaccination is advocated to counter the resurgence of the epidemic.

## Data availability statement

The raw data supporting the conclusions of this article will be made available by the authors, without undue reservation.

## Ethics statement

The studies involving human participants were reviewed and approved by Ethics Committee of Wuhan Union Hospital, Tongji Medical College, Huazhong University of Science and Technology. The patients/participants provided their written informed consent to participate in this study.

## Author contributions

XZ, BL, and TX designed and conceived the study. BL, TX, HW, and ZL performed the experiments. BL, TX, HW, ZL, XQ, SML, SHL, LF, LX, TW, XW, BZ, JW, DY, JL, and XZ enrolled patients and acquired the data. XF and LF assistant with sample collection. BL and TX analyzed the data, contributed to the charts and drafted the manuscript. XZ revised the manuscript. All authors contributed to the article and approved the submitted version.

## Funding

This study was supported by the National Science and Technology Major Project of China (92169121), the Applied Basic and Frontier Technology Research Project of Wuhan (2020020601012233), the Science and Technology Key Project on Novel Coronavirus Pneumonia, Hubei Province (2020FCA002), the Fundamental Research Funds for the Central Universities (2020kfyXGYJ016 and 2020kfyXGYJ028), and the Key Biosafety Science and Technology Program of Hubei Jiangxia Laboratory (JXBS001).

## Acknowledgments

We appreciate Shenzhen New Industry Biomedical Engineering Co., Ltd. for providing MAGLUMI SARS-CoV-2 antibodies assay kits.

## Conflict of interest

The authors declare that the research was conducted in the absence of any commercial or financial relationships that could be construed as a potential conflict of interest.

## Publisher’s note

All claims expressed in this article are solely those of the authors and do not necessarily represent those of their affiliated organizations, or those of the publisher, the editors and the reviewers. Any product that may be evaluated in this article, or claim that may be made by its manufacturer, is not guaranteed or endorsed by the publisher.
