# Supplementary material for: Robust humoral and cellular immune responses in long-term convalescent COVID-19 individuals following one-dose SARS-CoV-2 inactivated vaccination
Source: Front Immunol. 2022 Aug 1;13:966098. doi: 10.3389/fimmu.2022.966098 (PMC9377315; doi:10.3389/fimmu.2022.966098)
Supplement: Supplementary file 1 [file DataSheet_1.docx]

Supplementary Materials for

“Robust humoral and cellular immune responses in long-term convalescent COVID-19 individuals following one-dose SARS-CoV-2 inactivated vaccination”

**This file includes:**

Figures S1 to S4

Table S1 to S3

Figure S1


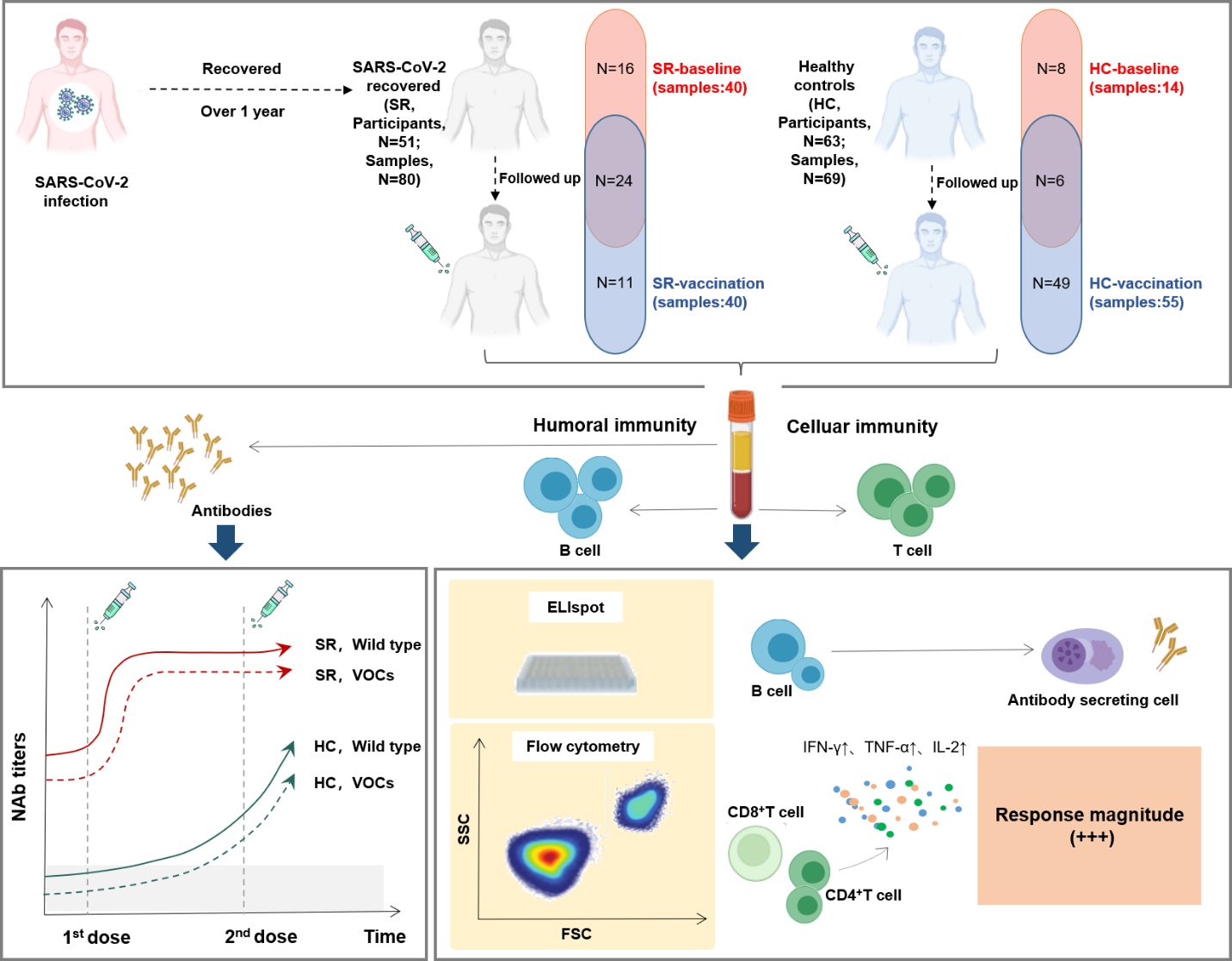


**Figure S1. Graphic abstract of study cohort** **and immune detection.**

51 SARS-CoV-2 long-term (over 1-year) recovered participants who were pre-vaccinated (SR-baseline) or had received inactivated vaccines (SR-vaccination) were consented and enrolled in our study. A total of 80 samples was collected and divided into SR-baseline (N=40) group and SR-vaccination (N=40) group. Across the two groups, 24 subjects were followed up longitudinally. 63 healthy subjects who were pre-vaccinated (HC-baseline) or had received inactivated vaccines (HC-vaccination) were enrolled as controls (HC). A total of 69 samples was collected and divided into HC-baseline (N=14) group and HC-vaccination (N=55) group. Across the two groups, 6 subjects were followed up longitudinally. Anti-S-RBD IgG and NAbs against the original SARS-CoV-2 (wild type, WT) and VOCs were measured via CLIA. Furthermore, SARS-CoV-2 RBD specific B cells response and antigen-specific T cells response to SARS-CoV-2 overlapping peptides were investigated through ELISpot and flow cytometry.

Figure S2


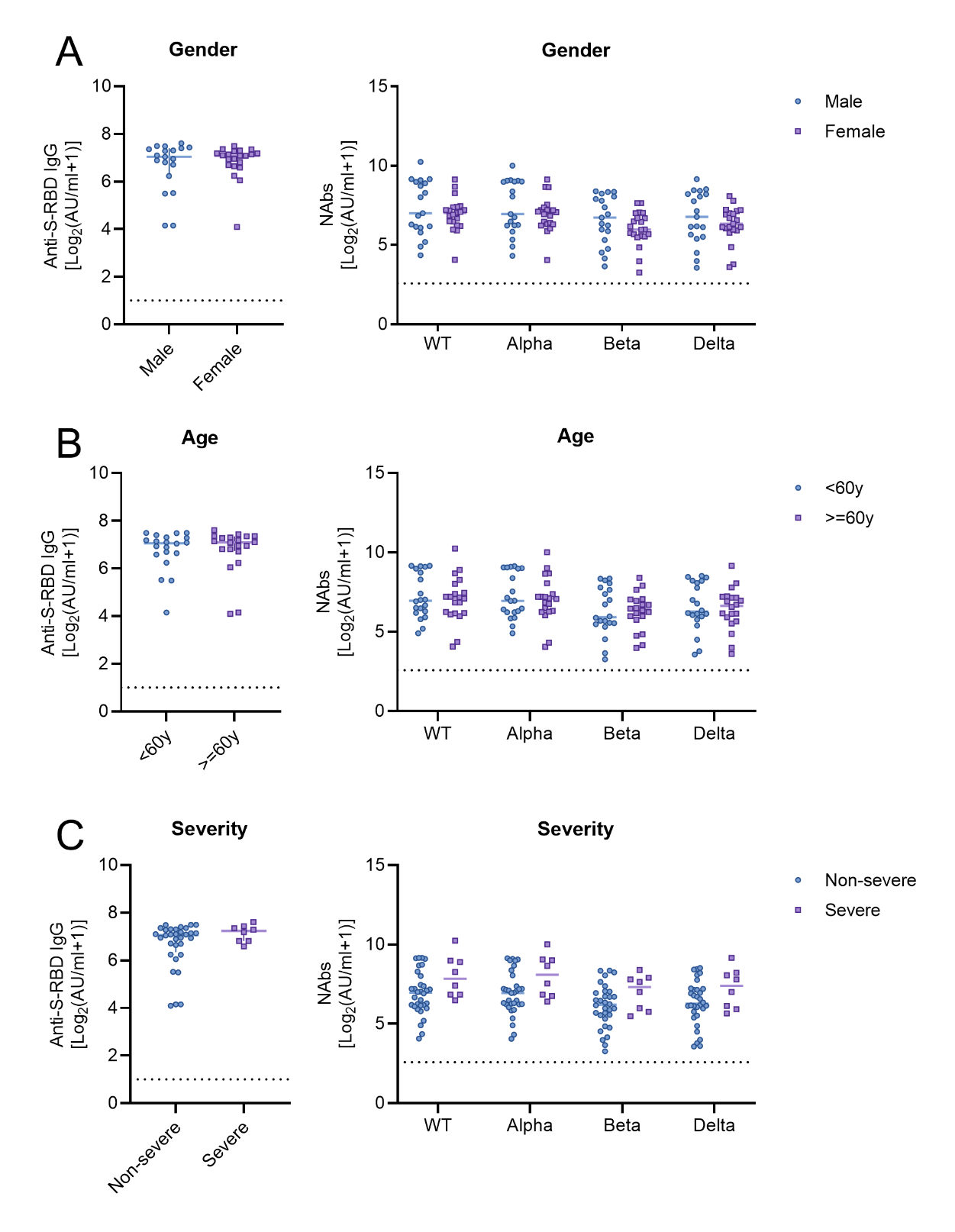


**Figure S2. The comparison of anti-S-RBD-IgG and NAbs stratified according to gender, age, and severity of illness SARS-CoV-2 recovered patients following booster vaccination.**

Figure S3


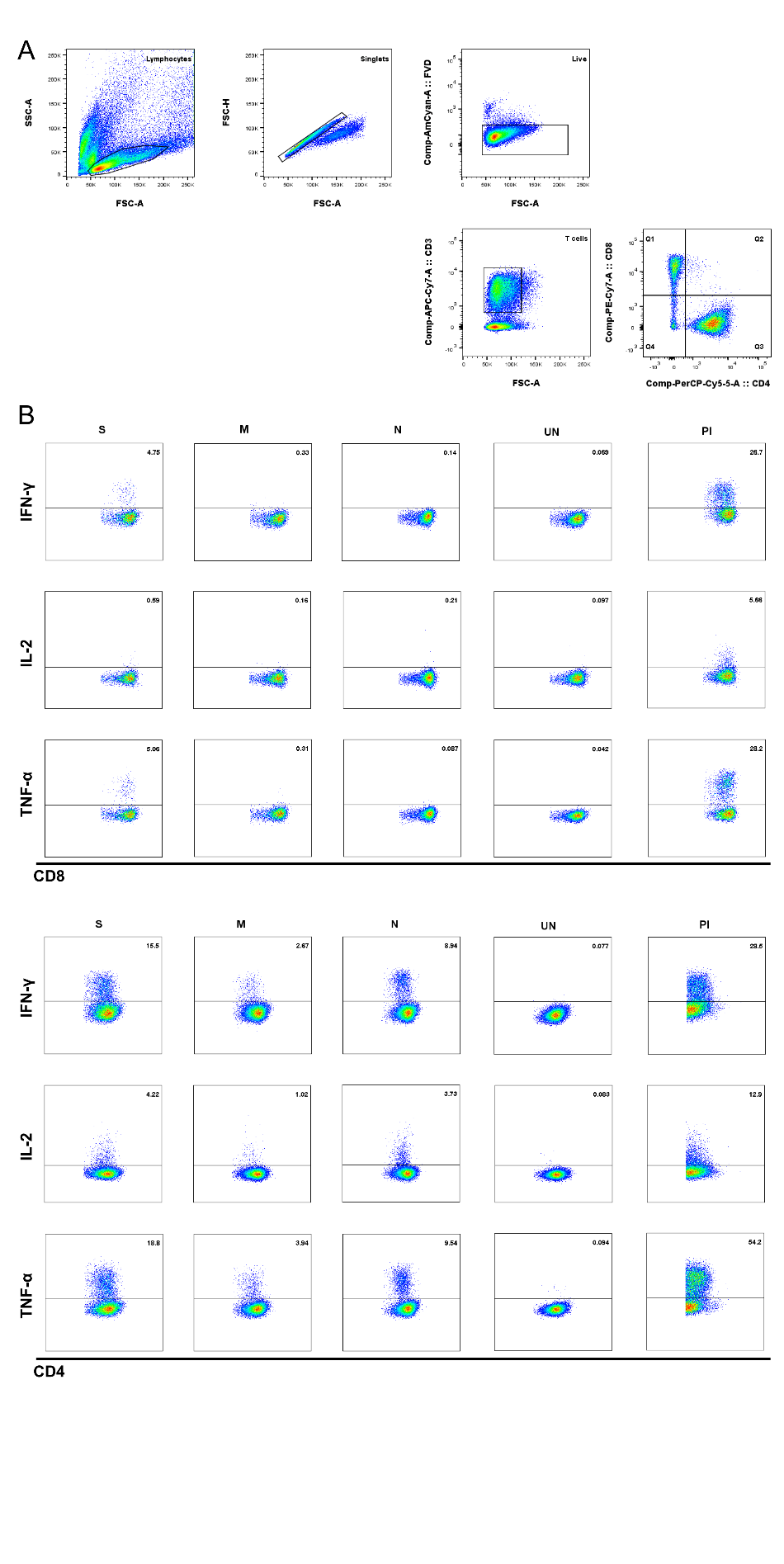


**Figure S3. The gating strategy of flow cytometry for intracellular cytokines (IFN-γ, IL-2, and TNF-α) production of CD4^+^ and CD8^+^ T cells responses specific to SARS-CoV-2 S, M, and N.** PI: positive control; UN: unstimulated control.

Figure S4


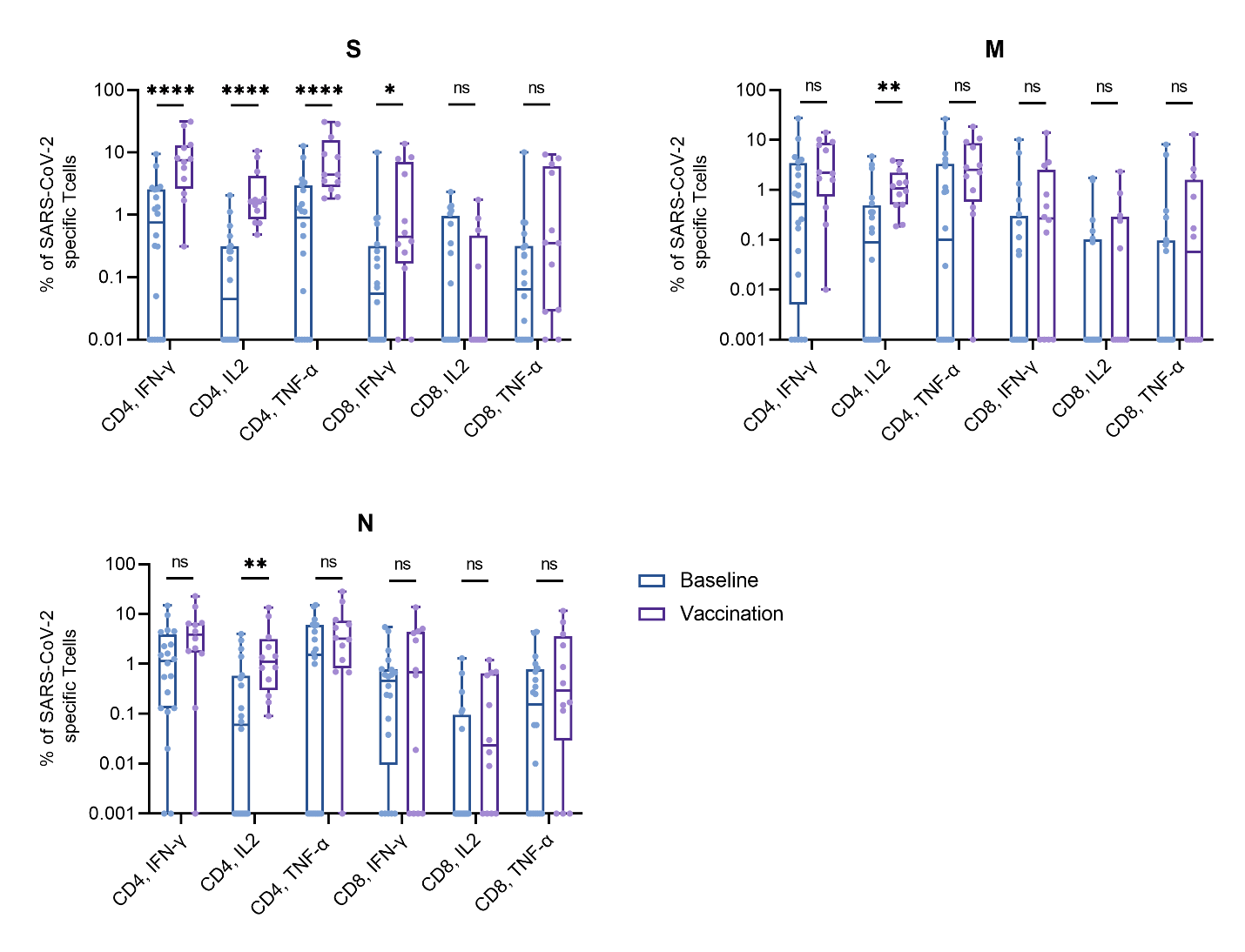


**Figure S4. Single effector cytokine (IFN-γ, IL-2, and TNF-α) expression of SARS-CoV-2-specific T cells responses in SARS-CoV-2 recovered patients.**

**Table S1.** **The demographic and vaccine-related information of the recruited participants ^a^**

|  | **HC-baseline**  **(N=14)** | **HC-vaccination**  **(N=55) ^b^** | **SR-baseline**  **(N=40)** | **SR-vaccination**  **(N=40) ^c^** |
| --- | --- | --- | --- | --- |
| **Age, [median (IQR)]** | 27 (25.5-27.5) | 30.5 (26-45) | 62 (52.5-67) | 58 (43-64) |
| **Sex** |  |  |  |  |
| Male [n (%)] | 2 (14.3%) | 13 (23.6%) | 20 (50.0%) | 19 (47.5%) |
| Female [n (%)] | 12 (85.7%) | 42 (76.4%) | 20 (50.0%) | 21 (52.5%) |
| **Time from disease onset, [median (IQR)]** | NA | NA | 357 (345-436) | 549 (518-592) |
| **Time from vaccination, [median (IQR)]** | NA | 30 (23.5-34) | NA | 44 (21-65.5) |
| **COVID-19 severity** |  |  |  |  |
| Severe [n (%)] | NA | NA | 11 (27.5%) | 8 (20.0%) |
| Non-severe [n (%)] | NA | NA | 29 (72.5%) | 32 (80.0%) |
| **Comorbidities, [n (%)]** | 1 (8.3%) | 3 (5.5%) | 23 (57.5%) | 20 (50.0%) |
| Hypertension | 1(8.3%) | 1 (1.8%) | 15 (37.5) | 7 (17.5%) |
| Diabetes | 0 (0%) | 0 (0%) | 7 (17.5%) | 3 (7.5%) |
| CVD | 0 (0%) | 1 (1.8%) | 2 (5.0%) | 0 (0%) |
| COPD | 0 (0%) | 0 (0%) | 2 (5.0%) | 1 (2.5%) |
| Chronic liver disease | 0 (0%) | 0 (0%) | 8 (20.0%) | 12 (30.0%) |
| Cancer | 0 (0%) | 0 (0%) | 2 (5.0%) | 1 (2.5%) |
| Other | 0 (0%) | 1 (1.8%) | 0 (0%) | 1. (0%) |

1. A total of 149 samples from 114 participants (51 SARS-CoV-2 recovered and 63 healthy) were collected and divided into four groups based on previous COVID-19 exposure and vaccination. HC-baseline: healthy individuals without vaccination, (N=14); HC-vaccination: healthy individuals with vaccination, (N=55)；SR-baseline: SARS-CoV-2 recovered individuals without vaccination, (N=40); SR-vaccination: SARS-CoV-2 recovered individuals with vaccination, (N=40).
2. All received two doses of inactivated vaccines and 6 participants had sequential samples pre- and post- vaccination.
3. Including 30 individuals following one-dose immunization and 10 individuals following two-dose immunization. 24 participants had sequential samples pre- and post- vaccination.

**Table S2.** Incidence of adverse reactions after vaccination in SARS-CoV-2 recovered and healthy individuals.

|  | **SARS-CoV-2 Recovered**  **(N=35)** | **Healthy control**  **(N=55)** | ***P value*** |
| --- | --- | --- | --- |
| **Total** **adverse reactions, [n (%)]** | 42.9% | 38.2% | 0.659 |
| **Local reactions, [n (%)]** | 31.4% | 32.7% | 0.898 |
| Injection-site pain and swelling | 31.4% | 32.7% | 0.898 |
| Injection-site induration | 0.0% | 1.8% | >0.999 |
| **Systematic reactions, [n (%)]** | 14.3% | 7.3% | 0.302 |
| Fever | 11.4% | 1.8% | 0.073 |
| Fatigue | 2.9% | 1.8% | >0.999 |
| Drowsiness | 2.9% | 0.0% | 0.389 |
| Dizziness | 2.9% | 3.6% | >0.999 |
| Rash | 2.9% | 0.0% | 0.389 |
| Myalgia | 0.0% | 1.8% | >0.999 |

**Table S3.** Multi-functionality of CD4+ and CD8+ T cell responses in SARS-CoV-2 recovered patients before and after vaccination.

|  | **S** |  |  | **M** |  |  | **N** |  |
| --- | --- | --- | --- | --- | --- | --- | --- | --- |
| No. of cytokines | Baseline  N (%) | Vaccination  N (%) |  | Baseline  N (%) | Vaccination  N (%) |  | Baseline  N (%) | Vaccination  N (%) |
| **CD4^+^ T cell** | |  |  |  |  |  |  |  |
| 0 | 7 (35.0) | **0 (0.0) *** |  | 8 (40.0) | **0 (0.0) *** |  | 8 (40.0) | 1 (8.3) |
| 1 | 3 (15.0) | 0 (0.0) |  | 3 (15.0) | 1 (8.3) |  | 1 (5.0) | 1 (8.3) |
| 2 | 3 (15.0) | 1 (8.3) |  | 4 (20.0) | 2 (16.7) |  | 5 (25.0) | 0 (0.0) |
| 3 | 7 (35.0) | **11 (91.7) *** |  | 5 (25.0) | **9 (75.0) *** |  | 6 (30.0) | **10 (83.3) *** |
| **CD8^+^ T cell** | |  |  |  |  |  |  |  |
| 0 | 6 (30.0) | 4 (33.3) |  | 11 (55.0) | 4 (33.3) |  | 8 (40.0) | 4 (33.3) |
| 1 | 6 (30.0) | 2 (16.7) |  | 6 (30.0) | 4 (33.3) |  | 3 (15.0) | 2 (16.7) |
| 2 | 5 (25.0) | 3 (25.0) |  | 2 (10.0) | 1 (8.3) |  | 6 (30.0) | 3 (25.0) |
| 3 | 3 (15.0) | 3 (25.0) |  | 1 (5.0) | 3 (25.0) |  | 3 (15.0) | 3 (25.0) |

* P<0.05
